# Transcriptomic Effects on the Mouse Heart Following 30 Days on the International Space Station

**DOI:** 10.3390/biom13020371

**Published:** 2023-02-15

**Authors:** Alicia L. Veliz, Lana Mamoun, Lorelei Hughes, Richard Vega, Bailey Holmes, Andrea Monteon, Jillian Bray, Michael J. Pecaut, Mary Kearns-Jonker

**Affiliations:** 1 Department of Pathology and Human Anatomy, Loma Linda University School of Medicine, Loma Linda, CA 92350, USA; 2 Department of Basic Sciences, Loma Linda University School of Medicine, Loma Linda, CA 92350, USA

**Keywords:** International Space Station, spaceflight, transcriptomics, cardiovascular system, RNA sequencing, adaptive response, signaling, oxidative stress

## Abstract

Efforts to understand the impact of spaceflight on the human body stem from growing interest in long-term space travel. Multiple organ systems are affected by microgravity and radiation, including the cardiovascular system. Previous transcriptomic studies have sought to reveal the changes in gene expression after spaceflight. However, little is known about the impact of long-term spaceflight on the mouse heart in vivo. This study focuses on the transcriptomic changes in the hearts of female C57BL/6J mice flown on the International Space Station (ISS) for 30 days. RNA was isolated from the hearts of three flight and three comparable ground control mice and RNA sequencing was performed. Our analyses showed that 1147 transcripts were significantly regulated after spaceflight. The MAPK, PI3K-Akt, and GPCR signaling pathways were predicted to be activated. Transcripts related to cytoskeleton breakdown and organization were upregulated, but no significant change in the expression of extracellular matrix (ECM) components or oxidative stress pathway-associated transcripts occurred. Our results indicate an absence of cellular senescence, and a significant upregulation of transcripts associated with the cell cycle. Transcripts related to cellular maintenance and survival were most affected by spaceflight, suggesting that cardiovascular transcriptome initiates an adaptive response to long-term spaceflight.

## 1. Introduction

The historic Apollo 11 mission to the moon stimulated a shift into a new era of research, merging biology and aerospace in an effort to achieve a greater understanding of the impact of spaceflight on the human body. Microgravity at low earth orbit (LEO), experienced aboard the International Space Station (ISS), causes changes in gravity-dependent fluid flow within the cardiovascular system [1,2,3]. Changes in cardiac output, heart rate and rhythm, blood pressure, and heart morphology have been reported following exposure to the spaceflight environment [4,5,6]. The low-dose radiation environment in space can also initiate structural damage at the DNA, cellular, and organ levels [2].

Ongoing studies using multi-omics suggest that the impact of spaceflight appears to vary depending on the model, the organ system, the differentiation status of the cells, and the length of spaceflight exposure [7,8]. Studies focused on the cardiovascular system have shown that human-induced pluripotent stem cell-derived cardiomyocytes (hiPSC-CMs) cultured on the International Space Station (ISS) responded to the spaceflight environment by altering the expression of genes involved in mitochondrial function [9]. In the cardiac tissue of mice, transcriptional changes induced after 15 days in space include the differential expression of genes related to the cell cycle, senescence, and redox balance [10]. Cardiac remodeling and alterations in the expression of proteasome and extracellular matrix genes occurred in the hearts of Drosophila after 30 days in spaceflight [11]. Little is known, however, about transcriptomic changes in mammalian hearts in vivo following 30 days of spaceflight. A better understanding of transcriptomic changes within the intact heart in space is important for long-term missions planned for the future. In this study, we provide a transcriptomic analysis of gene expression changes in the mouse heart after 30 days on the ISS.

## 2. Materials and Methods

### 2.1. Flight and Control Conditions

Female C57BL/6J mice aged 8–9 weeks old were obtained from The Jackson Laboratory (Bar Harbor, ME). Mice were shipped to the Kennedy Space Center (KSC) Space Life Sciences Laboratory (SLSL). Although there were several groups in this study, this report focuses on the transcriptomic changes in the heart when mice are housed in the spaceflight environment for 30 days. Mice were acclimatized for 1–2 weeks prior to the initiation of the study. Their living conditions were controlled for temperature and humidity and included a 12:12 h light–dark cycle. Food and water were provided ad libitum. During this adaptation period, the mice were adapted to the flight hardware (Rodent Research Hardware System). Mice flown on the International Space Station (ISS) and comparable ground control mice were maintained under identical conditions, including a single injection of saline. At 30 days, flight mice were transferred into the ISS Microgravity Sciences Glovebox (MSG), were euthanized via intraperitoneal (IP) injection of Ketamine/Xylazine (150/45 mg/kg) anesthesia followed by closed-cardiac puncture exsanguination and cervical dislocation. After removal of the spleen, mice were wrapped in foil, frozen and stored in the Minus Eighty Laboratory Freezer for ISS (MELFI) at −96 °C until sample return. NASA GLACIER and Cold Bag lockers were used to transport samples back to the ground following mission completion.

### 2.2. Tissue Collection and RNA Extraction for Sequencing

The frozen ground control and spaceflight mice were slightly thawed, dissected on ice, and the inferior two-thirds of the hearts were extracted. Cardiac tissue sections were stored in RNALater, RNAProtect, or were flash-frozen in liquid nitrogen. RNAProtect samples were stored at −20 °C. The RNALater and flash-frozen samples were stored at −80 °C. RNA was isolated from the hearts of three mice flown on the ISS and three hearts from comparable hardware- and age-matched ground control mice. The extraction of RNA from cardiac tissue was conducted using the miRNeasy Mini Kit (Qiagen, Valencia, CA, USA) following the manufacturer’s protocol. A total of 25 mg of cardiac tissue was homogenized with QIAzol (Qiagen) and centrifuged. The aqueous layer was extracted, and then filtered using RNeasy Mini Spin columns provided in the kit. RNA was eluted using 30 µL of RNase-free water. RNA quality was assessed using nanodrop and gel electrophoresis, and RIN >8 was verified prior to sequencing.

### 2.3. RNA Sequencing and Analysis

Poly(A) RNA sequencing was performed by LC Sciences (Houston, Texas) using RNA from three ground control mice and three spaceflight mice. The Poly(A) sequencing library was prepared using Illumina’s TrueSeq-stranded-mRNA sample preparation protocol. An Agilent Technologies 2100 Bioanalyzer was utilized to validate RNA integrity. An Agilent Technologies 2100 Bioanalyzer High Sensitivity DNA Chip was used to conduct quality control analyses and quantification of the sequencing library. Paired-end sequencing was performed on an Illumina’s NovaSeq 6000 system. To conduct transcript assembly, Cutadapt [12] and perl scripts in house were used to remove reads with adaptor contamination, low-quality bases, and undetermined bases. Sequence quality was verified using FastQC (http://www.bioinformatics.babraham.ac.uk/projects/fastqc/) version 0.10.1 (accessed on 01 September 2022). HISAT2 was utilized to map the genome of ftp://ftp.ensembl.org/pub/release-101/fasta/mus_musculus/dna/ v101 (accessed on 01 September 2022), while StringTie was used for transcript assembly [13,14]. The comprehensive transcriptome was generated from merged transcriptomes via perl scripts and gffcompare (https://github.com/gpertea/gffcompare/) (accessed on 01 September 2022). To conduct mRNA expression profiling, LC Sciences utilized StringTie and Ballgown (http://www.bioconductor.org/packages/release/bioc/html/ballgown.html) (accessed on 01 September 2022) [14]. StringTie specifically was used to calculate FPKM [14]. R package DESeq and edgeR were used to determine differentially expressed mRNAs [15,16]. A *p*-value of 0.05 and a fold change cutoff of 2 were chosen for this analysis. This subsequent data was analyzed u\sing Ingenuity Pathway Analysis (IPA) (Qiagen, https://www.qiagenbioinformatics.com/products/ingenuitypathway-analysis), (accessed 27 October 2022) [17]. RNA sequencing data was additionally uploaded to STRING and David Gene Ontology to determine significantly affected pathways and functions [18,19,20].

## 3. Results

### 3.1. Cardiac Transcriptome Alterations Induced by 30 Days in Spaceflight

**Figure 1:**
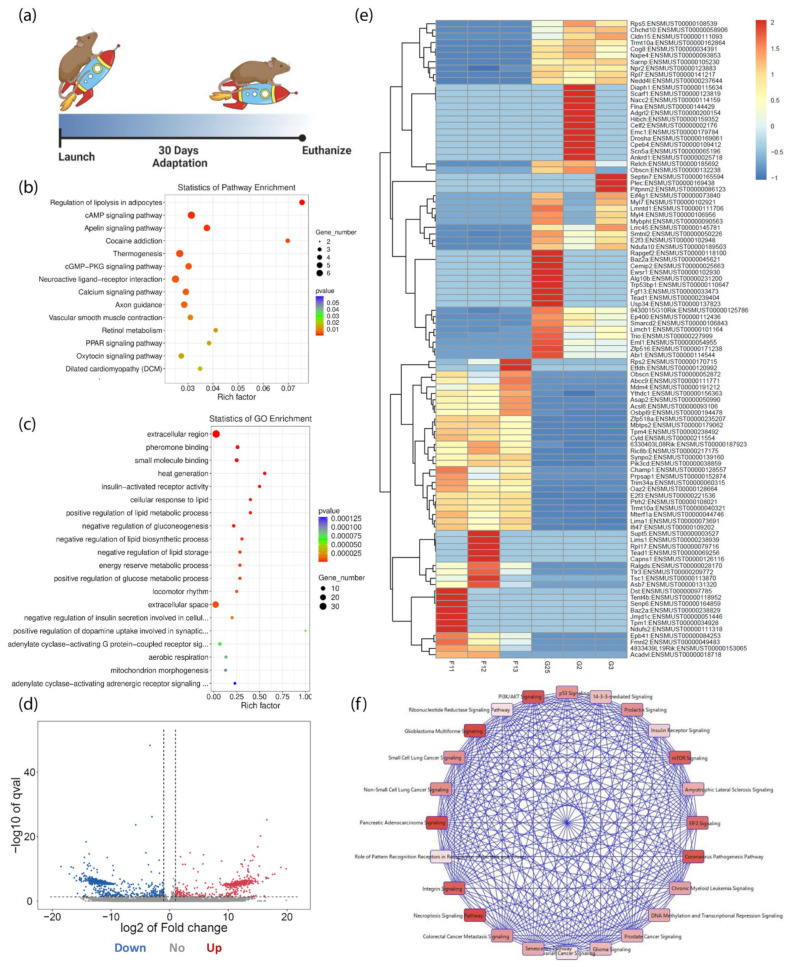
Transcripts Affected by Spaceflight. (**a**) Experimental timeline of spaceflight mice; (**b**) KEGG-pathway-enrichment scatterplot; (**c**) Gene ontology (GO)-enrichment scatterplot; (**d**) Volcano plot of transcript expression in the spaceflight group; (**e**) Heatmap of transcript expression levels showing the top 100 transcripts (*p* < 0.05) in spaceflight (F11, F12, F13) and control groups (G25, G2, G3); (**f**) IPA-generated pathway map relating to the top 100 significant transcripts. Each canonical pathway is colored proportionally to the *p*-value of a right-tailed Fisher’s test. A more saturated red indicates greater significance.

KEGG and Gene Ontology (GO) scatterplots were generated based on the top one hundred significantly (*p* < 0.01) expressed genes and transcripts in the spaceflight group (Figure 1a–c). Genes that function within the cAMP, cGMP-PKG, and calcium signaling pathways contributing to cell proliferation, cytoskeletal rearrangement, and survival were identified as enriched (Figure 1b). Spaceflight significantly altered the expression of Adcy9, Atp2a1, Adrb3, Nppa, Myl9, Adcy9, Grin2c, Ptgfr, Atp2a1, and Adrb3 in the cGMP-PKG and calcium signaling pathways. In the cAMP signaling pathway, five of six genes were downregulated, including Grin2c, Drd2, Nppa, Ppp1r1b, and Myl9, while Adcy9 was upregulated. This effect might be explained by the downregulation of Nppa expression. Nppa is an upstream regulator of Adcy9. GO analyses indicated that spaceflight has an impact on several molecular functions and biological processes (Figure 1c). A total of 1147 significantly regulated transcripts included 514 transcripts that were classified as upregulated (45%) and 633 transcripts that were downregulated (55%) in spaceflight (Figure 1d). The upregulated transcripts play a role in calcium, protein kinase A, ERK-MAPK, PI3K-Akt, senescence, cell cycle, and cAMP-mediated signaling. DAVID GO identified the cAMP signaling pathway, cGMP-PKG signaling pathway, and the regulation of actin cytoskeleton in the top ten most prevalent KEGG pathways from the 633 downregulated transcripts.

The top 100 most significant alterations in the transcriptome induced by spaceflight are shown in the heatmap (Figure 1e). The group of uniformly downregulated transcripts in the spaceflight group, namely transcripts Rps5 through Nedd4l, have enzymatic functions and roles in transcriptional regulation. Some were identified as ribosomal proteins. Similarly, in the group of uniformly upregulated transcripts, namely Ythdc1 through Ifi47, 11 transcripts contribute to cell survival and 10 transcripts have functional roles in cellular development, molecular transport, and protein trafficking. This upregulated transcript group is involved in cell-cycle checkpoint regulation, cellular assembly and organization, cell-to-cell signaling and interaction, p53 signaling, and cell survival. Within this large group of uniformly upregulated transcripts, Ric8b, Synpo2, Pik3cd, and E2f3 were of particular interest because they regulate the PI3K-Akt signaling pathway, cellular proliferation, the GPCR signaling pathway, and cell migration, respectively. From these top one hundred significant transcripts, a canonical pathway map was created to depict the most prevalent cellular signaling pathways. The cellular processes shown include the senescence pathway, PI3K-Akt signaling, p53 signaling, and E2F signaling (Figure 1f). The cellular processes most affected by stressful conditions in spaceflight are those that contribute to cellular maintenance and survival.

### 3.2. MAPK Signaling Is Activated by Spaceflight

Signaling pathways that impact cytoskeletal arrangement, CREB signaling, stress, cardiac hypertrophy, senescence, and other pathways involve MAPK signaling. The MAPK signaling pathway (also known as the Ras-Raf-MEK-ERK pathway or MAPKKK pathway) is a highly complex signaling cascade that involves six groups. Four subfamilies are the most well-known and include extracellular-signal-regulated kinases (ERK1/2), c-Jun NH_2_ terminal kinases (JNK1,−2,−3), p38 kinase, and MAPK (BMK/ERK5). This signaling pathway is activated through signal transduction from integrins and cell surface receptors, such as receptor tyrosine kinases and/or G-protein-coupled receptors.

**Figure 2:**
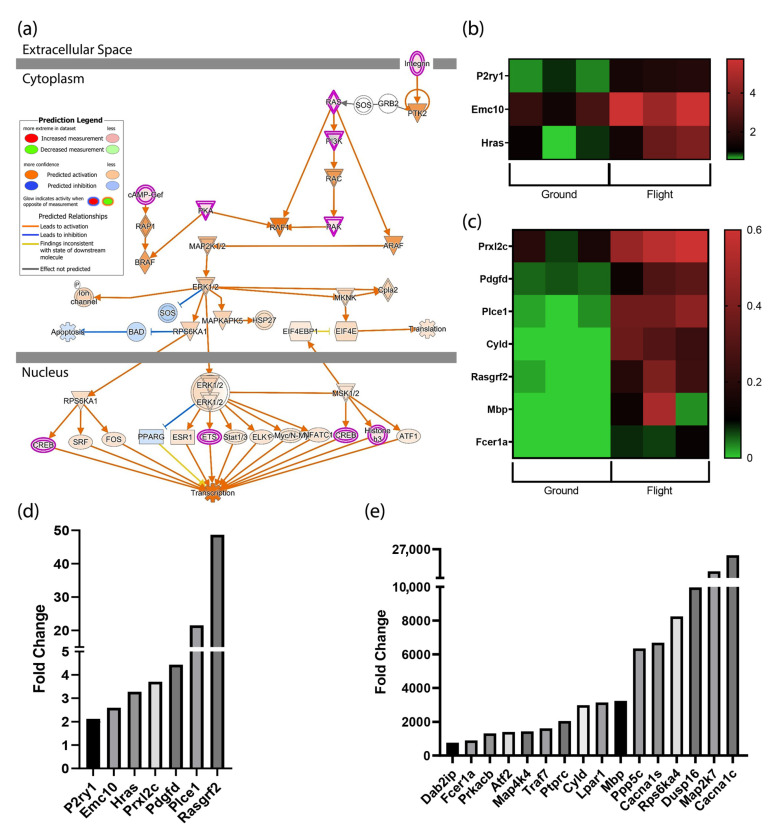
MAPK Signaling Is Upregulated in Spaceflight. (**a**) ERK/MAPK pathways are upregulated in the heart during spaceflight. IPA was used to identify mRNA transcripts predicted to be impacted in hearts from flight and ground control mice; (**b**,**c**) Heatmap comparing MAPK-associated transcript expression in flight and ground controls; (**d**,**e**) MAPK-related transcripts were significantly induced while in spaceflight (*p* < 0.001) vs ground controls.

One of the top three canonical pathways generated in IPA with a positive z-score was the ERK/MAPK signaling pathway (Figure 2a). ERK pathway activation involves the phosphorylation of p44 MAPK and p42 MAPK, also known as ERK1 and ERK2, ERK translocation to the nucleus, and the activation of several transcription factors. These transcription factors alter gene expression to promote growth, differentiation, and /or mitosis. As shown in Figure 2a, the upregulation of ERK1/2 and the inhibition of SOS and BAD are predicted to prevent apoptosis. Several transcripts induced by spaceflight and identified by IPA as significant (*p*-value 3.63 x 10^-3^) are shown in the heatmaps (Figure 2b,c).

Twenty-three transcripts related to the MAPK pathway were significantly induced. All twenty-three transcripts ranging from the lowest to highest level of expression are shown in Figure 2d,e. Seven transcripts were elevated within the range of 2 to ~50 fold (*p* < 0.001) (Figure 2d). Five of these seven transcripts regulate angiogenesis, proliferation, and play a role in preventing apoptosis. The final two transcripts, Plce1 and Rasgrf2, are involved in GTPase activity and calcium-regulated nucleotide exchange and were induced by 22 to 48 fold over transcript levels expressed in ground control mice. As shown in Figure 2e, the remaining 16 transcripts were also upregulated significantly. These transcripts were induced by 700 to 26,000+ fold, (*p* < 0.001). The most highly expressed transcripts are involved in signal transduction, mediating cell responses to proinflammatory cytokines, environmental stresses, ion channel subunits, serine/threonine-protein kinases, and protein phosphatases. The genes encoding these transcripts include Ppp5c, Rps6ka4, Map2k7, and Cacna1c.

### 3.3. Spaceflight Modifies Actin but Has Limited Effect on the ECM in the Heart

**Figure 3:**
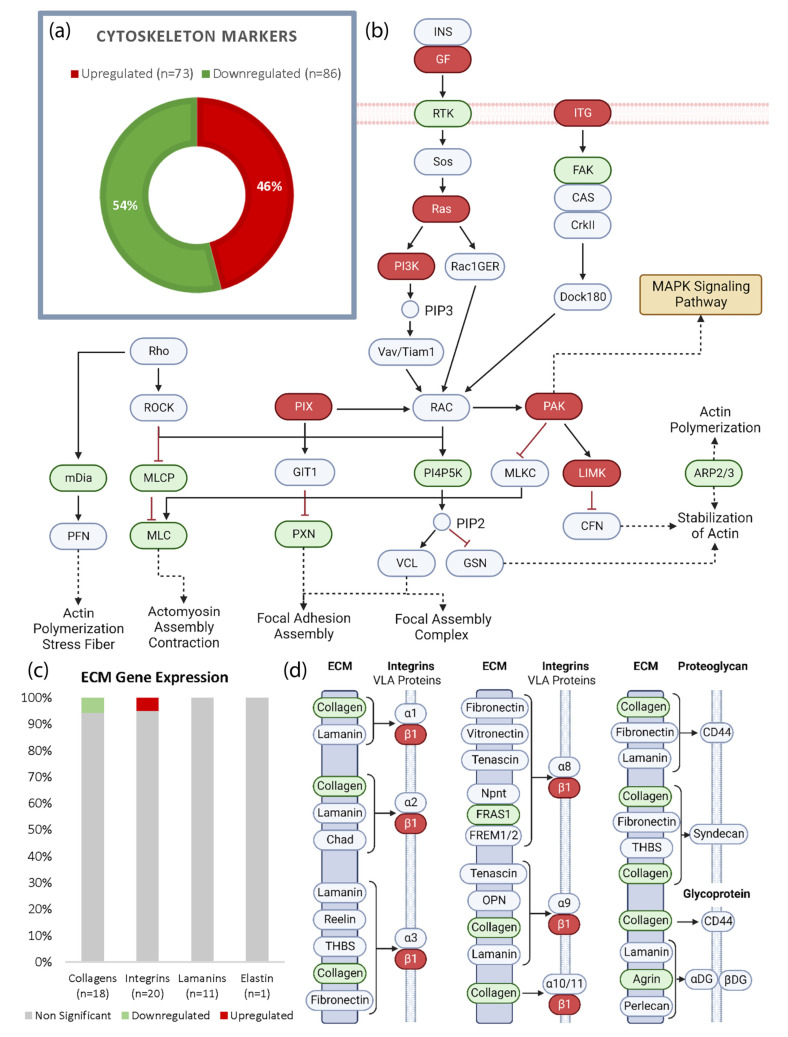
Spaceflight modifies actin but has limited effect on the ECM in the heart. (**a**) A total of 155 significantly modulated cytoskeleton-related transcripts were selected from the original transcript data received from LC Sciences. Of this subset, 86 were upregulated and 73 were downregulated. (**b**) This dataset was uploaded to DAVID Gene Ontology for subset analyses. The most significant KEGG analysis demonstrated there was regulation of the actin cytoskeleton in spaceflight with a downregulation of the products involved in focal adhesion, actin stabilization, actin polymerization, and actomyosin assembly (*p* = 8.8 x 10^-15^, Benjaminini = 1.6 x 10^-12^); (**c**) ECM components were analyzed for any changes induced by spaceflight, namely collagens, integrins, laminins, and elastin. Of these components, only two transcripts were significantly altered out of 50; (**d**) 37 significantly altered ECM-related transcripts were uploaded to DAVID GO for targeted analyses. KEGG analyses of ECM-binding products demonstrated limited interaction. FRAS1, collagen, and agrin were significantly downregulated and integrin beta 1 was significantly upregulated. Figures (**b**) and (**d**) were created using BioRender.com (accessed on 15 December 2022).

All significantly regulated transcripts were uploaded to DAVID Gene Ontology (DAVID GO) for functional enrichment and annotation analyses. Transcripts associated with the cytoskeleton (*p* = 1.0 x 10^-7^, Benjamini = 1.5 x 10^-5^) were significantly altered when comparing the spaceflight and control groups. To determine which aspects of the cytoskeleton were most heavily affected, 159 significant cytoskeleton-related transcripts were selected for target analyses. In total, 86 transcripts (54%) were downregulated and 73 transcripts (46%) were upregulated, shown in green and red, respectively (Figure 3a). The most significant finding from the KEGG analyses of this subset was regulation of the actin cytoskeleton (*p* = 8.8 x 10^-15^, Benjamini = 1.6 x 10^-12^). This information was used to generate a pathway demonstrating the dysregulation of the actin cytoskeleton (Figure 3b). Actin stabilization, actin polymerization, actomyosin assembly, and focal adhesion were all associated with downregulated transcripts, indicating a potential inhibition of these functions. The upregulation of PAK activates MAPK. Despite the significant dysregulation of the cytoskeleton, the evaluation of prominent extracellular matrix (ECM) components revealed that only 2 of 50 transcripts were altered (Figure 3c). Specifically, one collagen transcript, col4a5, was downregulated and one integrin, Itgb1, was upregulated. This reveals a predominantly unaltered transcriptome of products associated with ECM. Thrombospondins, fibronectin, vitronectin, and dystroglycan were also not altered by spaceflight in this model. To address the potential functional impact of all ECM transcripts that were affected, 37 significantly modulated ECM products were uploaded to DAVID GO. This allowed us to generate an ECM–receptor interaction chart based on KEGG analyses (Figure 3d). Collagen dysregulation has the potential to affect integrins involved with VLA proteins and proteoglycans. FRAS1 and agrin are of interest in multiple contexts as both play roles in organ development; however, in the context of ECM receptor binding, the downregulation of these transcripts can impact integrins and glycoproteins.

### 3.4. The PI3K-Akt Pathway Is Induced by Spaceflight

**Figure 4:**
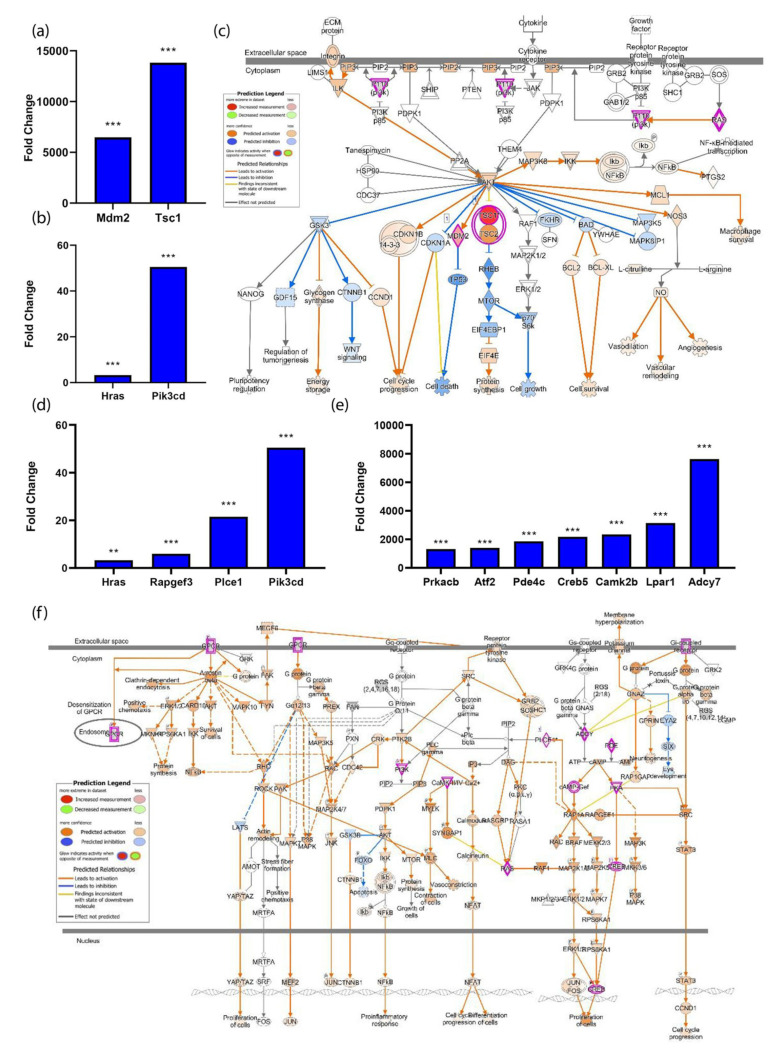
PI3K-Akt and G-Protein-Coupled Receptor Signaling is Upregulated in Spaceflight. (**a**,**b**) Significantly elevated transcripts in the spaceflight group associated with the PI3K-Akt pathway include the following: Mdm2, 6480-fold change; Tsc1, 13,830-fold change; Hras, 3-fold change; and Pik3cd, 50-fold change. (**c**,**f**) IPA-generated canonical pathways of PI3K-Akt and GPCR signaling. (**d**,**e**) Significantly upregulated transcripts in the spaceflight group associated with the GPCR pathway include the following: P2ry1, 2-fold change; Hras, 3-fold change; Rapgef3, 6-fold change; Plce1, 21-fold change; Pik3cd, 50-fold change; Prkacb, 1317-fold change; Atf2, 1405-fold change; Pde4c, 1861-fold change; Creb5, 2182-fold change; Camk2b, 2344-fold change; Lpar1, 3145-fold change; and Adcy7, 7631-fold change. ** = *p* < 0.01, *** = *p* < 0.001.

The PI3K-Akt pathway is one of the top five canonical pathways impacted by spaceflight with a *p*-value of 6.21 × 10^−3^. DAVID GO identified 18 genes associated with this pathway (Benjamini 1.8 × 10^−22^), where Mdm2, Tsc1, Hras, and Pik3cd transcripts were significantly elevated in expression with a fold change ranging from 3–13,830 (*p* < 0.001) (Figure 4a,b). Hras is an upstream promoter of Pik3cd, a component of the PI3K complex, which leads to the activation of cell survival, cell-cycle progression, cell proliferation, and protein synthesis (Figure 4c). G-protein-coupled receptor (GPCR) signaling was revealed as a significant canonical pathway (*p*-value = 1.22 × 10^−6^). Transcripts associated with GPCR signaling were induced by spaceflight, including P2ry1, Hras, Rapgef3, Plce1, Pik3cd, Prkacb, Atf2, Pde4c, Creb5, Camk2b, Lpar1, and Adcy7. These transcripts were upregulated in space, ranging from 2–7631 fold (*p* < 0.001) (Figure 4d,e). Multiple pathways downstream of GPCR signaling were activated or predicted to be activated by spaceflight (Figure 4f). These downstream pathways included YAP/TAZ, NFkB, AKT, IP3, DAG, MAPK, STAT3, CREB, RAS, and mTOR. The pathways predicted to be activated promote cell survival, proliferation, and enhance cellular response to oxidative stress (Figure 4f).

### 3.5. The Spaceflight Environment Activates Transcripts Associated with the Cell Cycle

**Figure 5:**
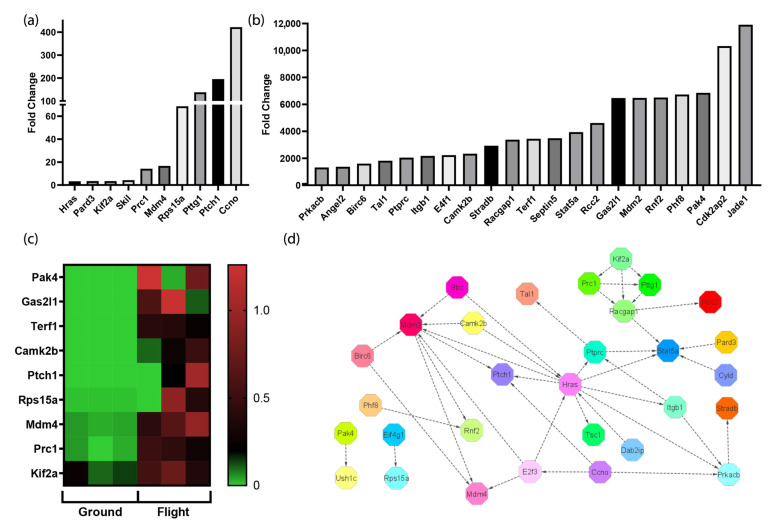
Cell-cycle transcripts are upregulated in spaceflight. (**a**,**b**) Differential transcript expression analyses revealed a statistically significant elevation of targets associated with activation of the cell cycle in spaceflight mice (*p* value < 0.001). (**c**) Heatmap showing differential expression of transcripts associated with proliferation. These transcripts are listed in descending order of fold change (~6000 to 3 fold). (**d**) A protein–protein interaction network with data generated in String demonstrates connections between the transcripts identified in (**a**) and (**b**).

Transcriptomic analyses comparing cardiac tissue samples collected from the space mice and the control mice maintained on the ground revealed elevated transcripts associated with the activation of the cell cycle in space. A fold change cutoff of 2.0 was applied to differentially expressed transcripts, resulting in the identification of thirty-one statistically significant transcripts that were found to be elevated. The fold changes ranged from 3 to over 6000 fold (*p* < 0.001) (Figure 5a,b). In murine embryonic stem cells, Cdk2ap2 is a regulator of cell renewal and is essential for survival during differentiation [21]. Pak4 plays a role in proliferation, cell migration, adhesion, and survival [22]. Phf8 regulates transcription factors necessary for cell-cycle progression and a lack of this transcript results in a prolonged G2 phase before mitosis [23]. The transcript with the highest fold change was Jade1, which activates transcription through the process of histone acetylation. Although the specific transcript encoding Jade1 has not been previously studied in the spaceflight environment, prior publications have reported that histone acetylation increases in microgravity [24,25]. A heatmap comparing the expression levels of transcripts associated with cell-cycle activation on the ground and in space is shown in Figure 5c. Figure 5d shows the network of interaction that ties the significantly elevated transcripts together. Hras and Mdm2 both activate and are activated by multiple transcripts. Hras activates cell progression and Mdm2 is known to induce proliferation and inhibit apoptosis [26,27]. The microgravitational environment enhances cell-cycle progression, coupled with cell survival in the cardiovascular system of mice.

### 3.6. Transcripts Associated with Cellular Senescence Are Not Significantly Elevated in the Heart

**Figure 6:**
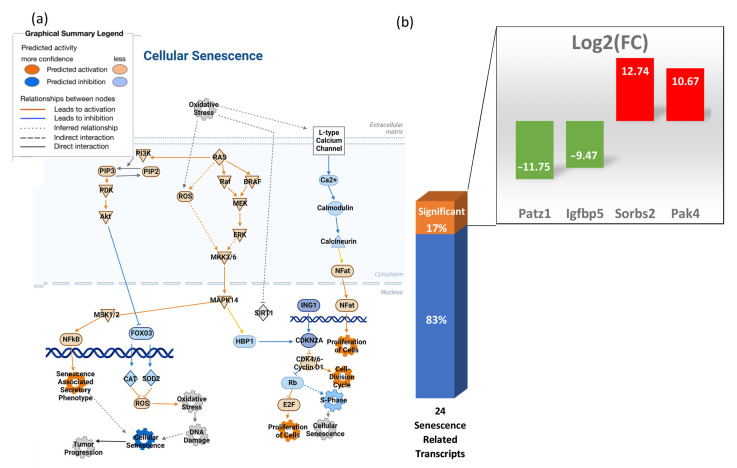
Cellular senescence is not predicted to be induced by spaceflight. (**a**) Pathway analysis was conducted using IPA, showing that cellular senescence is not likely to be induced in mouse hearts after 30 days in space; (**b**) 24 transcripts related to senescence were examined from transcriptomic data. Of these transcripts, only 4 variants were significant, with only 3 inducing senescence. Figure (**a**) was created using BioRender.com 2 December 2022.

Microgravity does not promote cellular senescence in the heart of space mice after long-term exposure. Figure 6a shows a pathway for cellular senescence that was created through IPA, comparing mice maintained on the ground and mice housed in space. Differential transcript expression values were uploaded to the IPA database for all significant genes related to the cell cycle. Using that analysis, a pathway was created to predict cellular senescence where reactive oxygen species (ROS) leads to oxidative stress, which causes DNA damage. According to IPA, ROS and oxidative stress molecules show low levels of predicted activation and DNA damage is not predicted. Ras activation, however, stimulates PI3k and MAPK. The downstream effect of this is the inactivation of senescence. Twenty-four transcripts related to senescence were analyzed (Figure 6b), of which only four were significantly altered by spaceflight, including Pak4, which was upregulated and functions to activate pro-survival pathways [22].

### 3.7. Oxidative Stress in the Heart

**Figure 7:**
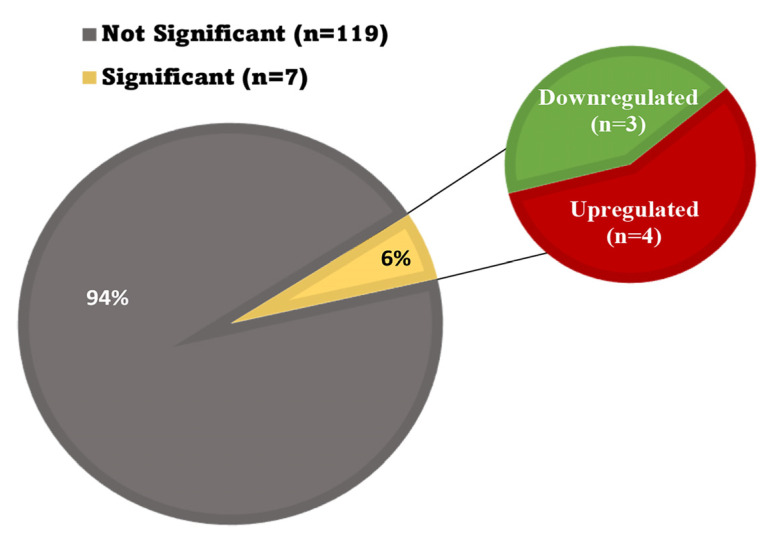
Prolonged spaceflight has a limited effect on oxidative stress in the heart. All genes associated with the oxidative stress pathway were uploaded for subset analyses in David Gene Ontogeny. Ninety four percent of transcripts that affect oxidative stress were not significantly changed by spaceflight after 30 days. Six percent of the total number of transcripts analyzed were significantly impacted by spaceflight (*p* ≤ 0.0005).

Most transcripts associated with oxidative stress were not significantly altered in the hearts of mice flown on the ISS for 30 days. As shown in Table 1, the fold change in the expression of 21 genes related to oxidative stress ranged from 0.22–1.62, with no significant difference in expression when comparing the ground control mice to the space mice (*p* > 0.05). A list of 126 transcripts associated with oxidative stress was created. The change in expression when comparing flight and ground conditions was only significant in seven of these genes (*p* ≤ 0.0005). The variants that were significant included 1600014C10Rik, Etfdh, Gpx1, and Ndufs2, which were upregulated, and Sirt1, Slc7a11, and Tor1a, which were downregulated. The expression of the remaining 119 genes in this group was unchanged as demonstrated in Figure 7.

## 4. Discussion

The increasing interest in space exploration and long-term spaceflight requires a better understanding of the reactive and adaptive changes that occur in various organ systems after exposure to the unique conditions of the space environment. Microgravity and radiation are known to induce alterations in gene expression; however, limited information has been reported defining the transcriptomic changes that impact organ system functions. This report provides a transcriptomic analysis of gene expression changes that occur in the mouse heart after 30 days of spaceflight aboard the ISS. To our knowledge, this study is one of the first to use an in vivo model to focus on the effects of long-term (30 day) spaceflight on gene expression, molecular pathways, and cellular changes occurring in the heart. Several signaling pathways and cellular processes were altered in a manner suggestive of cellular adaptation through mechanisms that promote survival.

The cytoskeleton adjusts to microgravity in a manner which follows the cellular tensegrity model [28], which purports the concept that gravitational change breaks the tension load on cytoskeleton and transmembrane proteins, consequently triggering reorganization [29]. Our studies on the mouse heart demonstrate that cytoskeleton breakdown and reorganization occur. We identified 83 transcripts that were significantly downregulated and 76 transcripts that were significantly upregulated, including integrins beta-1 (transmembrane proteins attaching the cytoskeleton to the extracellular matrix). Cytoskeletal changes may also be influenced by trophic changes in cardiomyocytes, affecting tension and load bearing. These alterations impact MAPK signaling pathways. Further studies conducted during extended periods of spaceflight (e.g., 6 months) are needed to determine whether cytoskeletal reorganization reaches an optimal point for cellular functioning. Interestingly, our model showed limited changes to the major components of the extracellular matrix, although the ECM undergoes modifications in spaceflight in the mouse lung and drosophila heart [11,30]. The impact of spaceflight on the ECM may differ according to organ, species, or length of exposure. An open versus closed cardiac system may result in significantly different adaptive responses.

Transcriptomic analyses reveal active cell proliferation linked to cell-cycle regulation. Known cell-cycle markers indicate that cells divide and survive in the heart. Recent studies show the enhanced proliferation of immune cells in mice undergoing microgravity exposure [31]. Infiltrating immune cells or endothelial cell activation may be contributing factors [32]. Enhanced proliferation could lead to cytoskeletal adaptations in spaceflight mice. Macrophages exhibit structural changes in their cytoskeletal arrangement, specifically impacting actin in response to microgravity [33].

MAPK signaling plays an essential role in cell-cycle activation, cell growth, differentiation, development, and survival [34,35]. MAPK signaling was predicted to be induced by spaceflight in the mouse heart. Prior studies using isolated cells flown on the ISS for 4–5 days reported an increased expression of small GTPases, Ras, and Rho, which coincides with our transcriptome results [36]. Ras/MAPK regulates stress signal responses and consequently elevates antioxidant transcripts [37]. Prxl2c transcripts were elevated in our findings, consistent with this interpretation. Peroxiredoxin-like 2C (Prxl2c) is predicted to enable antioxidant activity and functions in the positive regulation of ERK1/2 and in the regulation of glycolytic processes. MAPK has also been reported to be activated in the mouse brain by microgravity [38].

The cytoprotective role of MAPK is one of the many downstream effects of GPCR and PI3K pathway activation [34,39]. It is well understood that the activation of GPCR signaling and the PI3K-AKT pathway leads to cellular survival, cell-cycle progression, and, more specifically, cardiomyocyte survival and cardiovascular homeostasis [40,41,42]. Previous studies in other models focusing on the role of PI3K in cardiac health have revealed the role of this pathway in eliciting protective changes following physiological stress [42,43,44,45,46]. The impact of spaceflight on this signaling pathway in a cardiac model in space, however, is not well understood. Cultured cells exposed to spaceflight conditions have shown an increase in G-protein alpha subunit activation and elevated MAPK signaling activity [36,47]. Under short-term simulated microgravity conditions, decreased PI3K and AKT expression and higher apoptotic rates were reported in cultured cells [48]. Detrimental physiological changes have been associated with the downregulation of the PI3K-Akt signaling pathway [49]. Our study revealed a significant upregulation of multiple transcripts that comprise the GPCR and PI3K-Akt signaling pathways in cardiac tissue following 30 days in space. Our findings suggest that cellular survival and adaptation occur through the upregulation of the GPCR and PI3K-Akt pathways as a response to long-term exposure to the space environment. The activation of the GPCR and PI3K-Akt signaling pathways may function in a protective capacity during spaceflight to counter the effects of the physiologically stressful environment. To our knowledge, little is known about the effect of the space environment on the GPCR and PI3K-Akt signaling pathways in the cardiovascular system in live animals subjected to long-term spaceflight. Thus, a better understanding of the molecular changes within the cardiovascular system that allow for adaptation to the space environment are necessary to prepare for future long-term space travel.

Surprisingly, oxidative stress was not significantly induced in the heart at 30 days. Mitochondria are the source of reactive oxygen species (ROS), and microgravity has been shown to activate its production. The imbalance of ROS versus the processes that prevent oxidative stress can cause the mitochondria to become dysfunctional [50]. Most of the transcripts involved in the regulation of oxidative stress were not affected in our model; only 7 of 126 transcripts examined showed a significant change in expression. Five of these transcripts offered cell protection and only two were impacted in a manner that would be expected to promote ROS production. These transcripts were Ndufs2, which forms part of the core of NADH dehydrogenase complex I, and Etfdh, which is part of the electron transport chain. Both have the potential to affect ROS levels in the mitochondria [51]. However, it is notable that although these significant transcripts can increase ROS levels, they may not be sufficient independently. Because it is well accepted that microgravity can increase the concentration of reactive oxygen species [52], it is possible that the oxidant nature of ROS further activates Ras/MAPK cascades that respond to stress signals and create a balanced system through the increased transcription of antioxidants [37]. This could explain why a significant upregulation of Gpx1 was noted in our study. Gpx1 increases glutathione levels to aid in oxidative defense [53]. In addition to activating the antioxidative response, MAPK functions to induce cellular homeostasis in conjunction with processes such as the ubiquitin-proteasome system, which is known to regulate and degrade proteins within the MAPK pathway [54]. The proteasome units, once activated, can begin degrading not only the MAPK transcripts, but also the oxidized proteins which are typically found in excess during oxidative stress [55]. Oxidative stress has been shown to increase due to microgravity [52]. The ability to manage oxidative stress may be influenced by the time of exposure or the organ system studied. Research on mice and mouse-derived cells in spaceflight has often focused on organs other than the heart. This includes the brain and skeletal system, although there was a recent publication that demonstrated select genes associated with oxidative stress in the heart were elevated in shorter-term spaceflight exposure [10,56,57]. Interestingly, the expression of these genes was not elevated at 30 days. We speculate that our experimental data could be due to acclimatization in the heart during the extended time mice were exposed to microgravity.

Cellular senescence may occur as a consequence of exposure to the stress of the spaceflight environment. Senescence occurs when cells enter cell-cycle arrest [58]. Within the mouse heart, our findings demonstrate that cell-cycle progression was not impeded by spaceflight, oxidative stress was managed, and cellular senescence was not predicted to occur by transcriptomic analyses. Stress mechanisms activate senescence, yet our data show that transcriptomic changes associated with oxidative stress were not significantly induced after 30 days in space. This may, in part, explain why senescence was not predicted to be activated in the heart. It is also possible that the young age of the mice utilized in our study may have obscured senescence-related changes [59]. Spaceflight-related senescence may be better observed in older mice. Additionally, our study was limited to the use of female mice. In humans, female astronauts suffer different cardiac effects compared to their male counterparts [60]. Studies planned for the future should be designed to include animals of both sexes in order to determine whether sex-based disparities occur in responses to the spaceflight environment. Sequential timepoint analyses would also be beneficial in order to provide insight regarding the ways in which the length of exposure to the spaceflight environment influences adaptive and reactive changes in the heart.

Based on our findings, we propose that 30 days in spaceflight provides enough time for the initiation of an adaptive response to the space environment to occur within the murine heart. Out of 86,406 analyzed transcripts, only 1147 transcripts (1.3%) were significant in the flight group, suggesting that by 30 days the heart has successfully adapted to the stressful space conditions. Some cellular processes that may contribute to adaptation and survival are the PI3K-Akt pathway, GPCR signaling pathway, and MAPK signaling pathway. Transcriptomic analyses predicted the activation of these pathways in the flight group. The ability of the heart to adapt to the space environment after long-term spaceflight is one possible explanation for the lack of differences in ROS and oxidative stress when comparing the flight and ground control groups in our study. Additionally, over time, adaptation can shift the focus from survival to growth, away from senescence, and towards cell proliferation. Survival in space may be facilitated by the adaptive nature of the transcriptome.

## Figures and Tables

**Table 1:** Differential Gene Expression Changes in Oxidative Stress Markers after Spaceflight.

Gene Symbol	Gene Name	Fold Change
Cat	Catalase	1.13
Fancc	Fanconi anemia, complementation group C	0.82
Gpx1	Glutathione Peroxidase 1	0.78
Gpx3	Glutathione Peroxidase 3	0.27
Gsr	Glutathione reductase	1.00
Gstp1	Glutathione S-transferase, pi 1	0.83
Ncf2	Neutrophil cytosolic factor 2	0.78
Nox1	NADPH oxidase 1	0.65
Nox4	NADPH oxidase 4	0.49
Prdx1	Peroxiredoxin 1	1.06
Prdx6	Peroxiredoxin 6	1.05
Prnp	Prion protein	1.02
Ptgs2	Prostaglandin-endoperoxide synthase 2	0.22
Slc41a3	Solute carrier family 41, member 3	1.62
Sod1	Superoxide dismutase 1	0.87
Sod2	Superoxide dismutase 2	1.05
Sod3	Superoxide dismutase 3	0.78
Tpo	Thyroid peroxidase	1.00
Txnip	Thioredoxin interacting protein	0.88
Txnrd3	Thioredoxin reductase 3	0.98
Xpa	Xeroderma pigmentosum, complementation group A	0.72

Listed in the table are 21 genes that have been linked to the oxidative stress pathway. These genes show no significant regulation in stress response after 30 days of spaceflight, as defined by a *p*-value greater than 0.05.

## Data Availability

The transcriptomic data is freely available and can be accessed in the NCBI Gene Expression Omnibus (GEO; http://www.ncbi.nlm.nih.gov/geo/ (accessed on 25 January 2023) under accession number GSE223803.
